# Automated analysis of small animal PET studies through deformable registration to an atlas

**DOI:** 10.1007/s00259-012-2188-7

**Published:** 2012-07-21

**Authors:** Daniel F. Gutierrez, Habib Zaidi

**Affiliations:** 1 Division of Nuclear Medicine and Molecular Imaging, Geneva University Hospital, 1211 Geneva 4, Switzerland; 2 Geneva Neuroscience Center, Geneva University, 1205 Geneva, Switzerland; 3 Department of Nuclear Medicine and Molecular Imaging, University Medical Center Groningen, University of Groningen, 9700 RB Groningen, Netherlands

**Keywords:** PET/CT, Small animals, Quantification, Deformable registration, Atlas

## Abstract

**Purpose:**

This work aims to develop a methodology for automated atlas-guided analysis of small animal positron emission tomography (PET) data through deformable registration to an anatomical mouse model.

**Methods:**

A non-rigid registration technique is used to put into correspondence relevant anatomical regions of rodent CT images from combined PET/CT studies to corresponding CT images of the Digimouse anatomical mouse model. The latter provides a pre-segmented atlas consisting of 21 anatomical regions suitable for automated quantitative analysis. Image registration is performed using a package based on the Insight Toolkit allowing the implementation of various image registration algorithms. The optimal parameters obtained for deformable registration were applied to simulated and experimental mouse PET/CT studies. The accuracy of the image registration procedure was assessed by segmenting mouse CT images into seven regions: brain, lungs, heart, kidneys, bladder, skeleton and the rest of the body. This was accomplished prior to image registration using a semi-automated algorithm. Each mouse segmentation was transformed using the parameters obtained during CT to CT image registration. The resulting segmentation was compared with the original Digimouse atlas to quantify image registration accuracy using established metrics such as the Dice coefficient and Hausdorff distance. PET images were then transformed using the same technique and automated quantitative analysis of tracer uptake performed.

**Results:**

The Dice coefficient and Hausdorff distance show fair to excellent agreement and a mean registration mismatch distance of about 6 mm. The results demonstrate good quantification accuracy in most of the regions, especially the brain, but not in the bladder, as expected. Normalized mean activity estimates were preserved between the reference and automated quantification techniques with relative errors below 10 % in most of the organs considered.

**Conclusion:**

The proposed automated quantification technique is reliable, robust and suitable for fast quantification of preclinical PET data in large serial studies.

## Introduction

One of the most significant advantages of positron emission tomography (PET) over other forms of functional imaging techniques is its capability to quantify absolute regional radiotracer concentration. Therefore, PET can generate quantitative dynamic images of regional radiotracer uptake, resulting in regional measurements of kinetic parameters. Quantification provides the direct relationship between the activity concentration measured in vivo in organs/tissues and the underlying physiological or pharmacokinetic processes occurring in the structure of interest [1]. It correlates the variation over time of the activity concentration to physiologically relevant quantitative parameters. In preclinical studies, quantitative assessment of tissue uptake permits better management of the therapy for an individual animal model and eventually enables assessment of overall response to a therapy in a population of transgenic animals [2]. However, quantitative PET is challenged by the need for appropriate compartmental or kinetic models to derive estimates of such parameters from dynamic PET measurements of regional activity concentrations, which is difficult to achieve in a clinical setting. Moreover, to take full advantage of the quantitative capabilities of PET imaging, patient-specific correction of background and physical degrading factors must be performed.

Automated quantitative assessment of metabolic PET data is appealing given its usefulness in terms of facilitating experimental molecular imaging investigations, since it can reduce variability across institutions and may improve the reliability of image interpretation independent of reader experience. For example, the development of tracer-specific small animal PET probabilistic atlases [3] correlated with anatomical (e.g. MRI) templates enabled automated volume of interest (VOI) or voxel-based analysis of small animal PET data with minimal end-user interaction [4]. One such software tool was developed by Kesner et al. [5] to enable assessment of the biodistribution of PET tracers using small animal PET data. This is achieved though non-rigid registration of a digital mouse model with the animal PET image set followed by automated calculation of tracer concentrations in 22 predefined VOI representing the major organs and remaining whole body. The development of advanced anatomical models including both stylized and more realistic voxel-based mouse [6–8] and rat [9, 10] models obtained from serial cryosections or dedicated high-resolution small animal CT and MRI scanners will certainly help to support ongoing research in this area [11].

Our objective in this work is to develop and assess the performance of atlas-guided automated analysis of small animal PET data based on computerized anatomical models and retrospective registration-guided methods enabling correct localization and accurate quantification of molecular targets. Our approach is different from the one adopted by Kesner et al. [5] in the sense that we are aiming at developing a fully automated analysis procedure which does not require user interaction and does not rely on the use of external fiducial markers or internal landmarks.

A substantial number of techniques have been proposed to achieve the goal of multimodal medical image registration [12, 13]. However, image registration algorithms widely used in clinical studies have not been well characterized in the small animal setting. A number of investigators focused on the utility of popular image registration techniques in various scenarios and using different imaging technologies and reported various degrees of success [14–22]. Some techniques rely on the use of external fiducial markers or specially designed hardware devices [23] to aid the registration process, whereas other approaches rely on fully automated algorithms that do not involve user interaction. Current state-of-the-art image registration techniques allow for automatic image registration through a rigid body transformation, thus ignoring organ deformation. There has also been noticeable progress in non-rigid registration algorithms that can compensate for perceived organ deformation for different imaging modalities or align images from different subjects [24]. However, despite progress made during the last few years, many image registration problems, particularly for small animal imaging, remain unsolved, and this is likely to continue to be an active field of research in the future [25].

The rationale of the automated 3-D image registration procedure used in this work to register PET images of the actual animal to an atlas for automated analysis is that it should be easier to find the correct alignment of anatomical CT images of the animal and those of the atlas than it is for noisy low-resolution PET images. This is achieved through non-rigid registration based on a multi-resolution approach. The transformation parameters obtained from CT to CT registration are then applied to corresponding PET images followed by automated quantitative analysis of tracer uptake in predefined regions.

## Materials and methods

### Mouse atlas

**Fig. 1:**
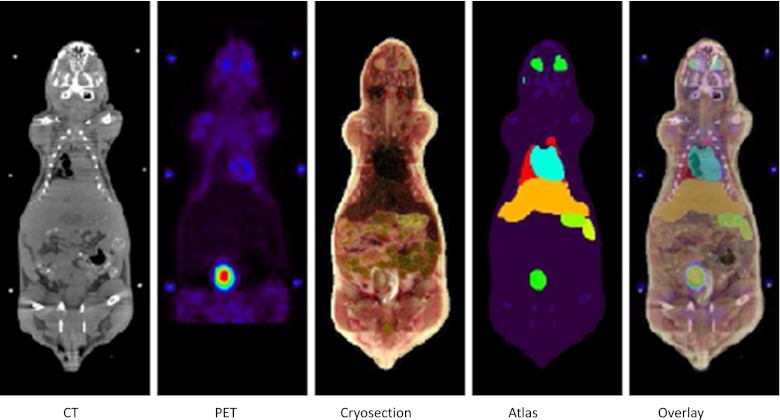
Spatially registered multimodality images for a coronal slice through the Digimouse model. From *left to right*: X-ray CT, PET, cryosection, segmented atlas and overlay of the atlas onto cryosection

The Digimouse atlas [7], composed of preregistered slices of PET, CT and cryosection images and the corresponding atlas, was used in this work. The latter results from a segmentation of 21 VOI including the skin, skeleton, eye, medulla, cerebellum, olfactory bulbs, external cerebrum, striatum, heart, the rest of the brain, masseter muscles, lachrymal glands, bladder, testis, stomach, spleen, pancreas, liver, kidneys, adrenal glands and lungs. Two versions of this data set are available: processed images coded in 8-bit (256 grey levels) and raw images coded on 32-bit floating-point format. The use of processed images would lead to loss of information during the registration process owing to the lower dynamic range. For this reason, we have reprocessed the raw images to obtain a data set similar to the processed one but with an isotropic voxel having a size of 0.2 mm and with coding to match typical CT and PET images in terms of dynamic range (16 and 32 bits, respectively) and scale (HU for CT). A representative coronal slice of the resulting images is shown in Fig. 1.

### Mouse PET/CT studies

#### Experimental studies

**Fig. 2:**
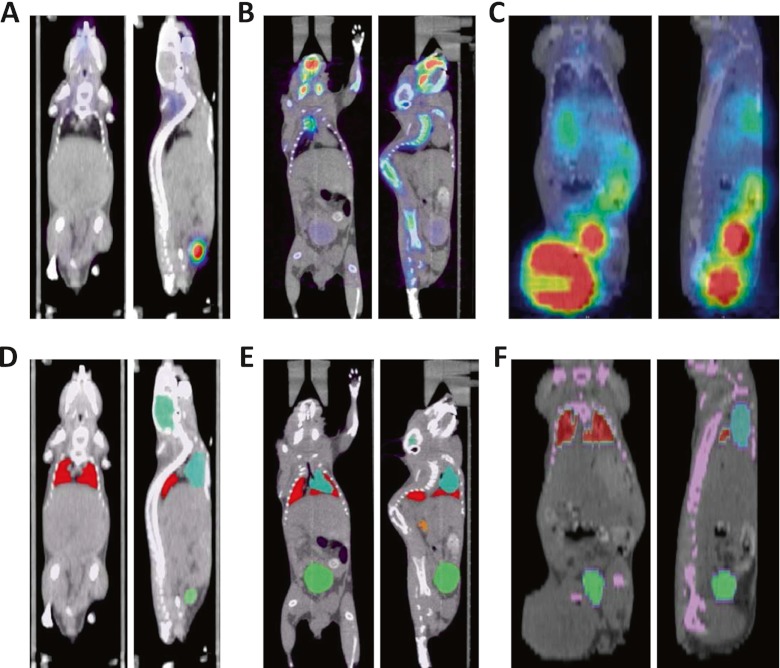
Example of coronal and sagittal slices of PET/CT studies (**a-c**) and overlay of the atlas onto CT images (**d-f**) of the experimental mouse studies acquired using ^18^F-FDG (*left*), ^18^F-NaF (*middle*) and bispecific antibody labelled with ^68^Ga (*right*) radiotracers

The sample of laboratory animals included in this work is composed of eight mouse PET/CT studies acquired using three different radiotracers with segmentations performed in our laboratory. Most of these mice had tumour xenografts producing large morphological deformations that increased the difficulty of the image registration process and challenges of automated quantification tasks (Fig. 2):

- One ^18^F-fluorodeoxyglucose (FDG) mouse acquired on the FLEX Triumph™ preclinical PET/CT scanner (Gamma Medica-Ideas, Northridge, CA, USA) [26], consisting of 16-bit CT images of 256 × 256 × 512 voxels of 0.17 × 0.17 × 0.17 mm^3^, and 32-bit PET images of 256 × 256 × 256 voxels of 0.4 × 0.4 × 0.4 mm^3^.
- Four mouse studies (three ^18^F-FDG and one ^18^F-NaF) acquired on the Siemens MicroFocus scanner kindly provided by the Crump Institute at UCLA composed of CT images of 256 × 256 × 496 voxels of 0.2 × 0.2 × 0.2 mm^3^ coded in 16 bits and PET images of 128 × 128 × 95 voxels of 0.4 × 0.4 × 0.8 mm^3^ coded in 32 bits.
- Three mice from the Applied Sciences Laboratory at Uppsala acquired for 60 min in list-mode format on the FLEX Triumph™ preclinical PET/CT scanner (Gamma Medica-Ideas, Northridge, CA, USA), composed of CT images of 240 × 240 × 63 voxels of 0.25 × 0.25 × 1.175 mm^3^ coded in 16 bits and PET images of 240 × 240 × 63 voxels of 0.25 × 0.25 × 1.175 mm^3^ coded in 16 bits. These three mice were injected with a bispecific antibody labelled with ^68^Ga.

Since our approach relies on 3-D image registration, segmented images of experimental animal images are needed for objective assessment of the accuracy of the image registration algorithm. Therefore, corresponding images were segmented into seven regions/organs: brain, lungs, heart, kidneys, bladder, skeleton and “remaining organs & skin”. Segmentation of CT images was performed using the ITK-SNAP software [27], which provides flexible tools for semi-automated medical image segmentation. The choice of segmented organs/regions was dictated by the need to have organs located at various portions of the mouse provided that soft tissue contrast of CT images is high enough to enable reliable segmentation.

The size and organ volume variation of this sample of laboratory mice is shown in Table 1. The sizes (e.g. cephalad-caudad length) were calculated from mouse segmentations by calculating the number of transaxial slices containing the mouse and multiplying this number by the slice thickness. The calculation of organ volumes was performed by counting the number of voxels belonging to each organ (example voxels labelled as brain) and multiplying it by the voxel volume. These measurements relied on complete whole-body mouse images for all mice except three where the assessment did not include the head since this part was outside the field of view.

**Table 1:** Physical characteristics of mice used in the experimental group

		Minimum	Maximum	Mean	SD
Whole-body dimensions (mm)	Cephalad-caudad	84.830	97.160	92.640	5.200
	Ventral-dorsal	19.720	31.750	25.310	4.190
	Lateral	23.970	40.000	32.120	4.460
Volume (ml)	Brain	0.314	0.358	0.344	0.018
	Heart	0.188	0.367	0.267	0.051
	Lungs	0.280	0.515	0.380	0.082
	Kidneys	0.154	0.418	0.291	0.086
	Bladder	0.021	0.458	0.172	0.141
	Skeleton	1.332	1.904	1.697	0.247
	Remaining organs & skin	12.373	20.761	17.843	3.342
	Total	15.345	23.865	20.889	3.489

#### Simulated studies

More elaborate analysis under controlled conditions (ground truth known) was carried out using 17 simulated PET/CT data sets. The simulated mouse sample was generated using the Moby digital mouse model [6], which creates two 3-D voxelized phantoms, one containing the activity map and the second the attenuation map (μ-map) at 511 keV. These two voxelized phantoms take into account respiratory and cardiac motions during an acquisition of 120 s.

Mice simulations were performed to produce a normal mouse sample and to complement the relatively small experimental mouse sample size by increasing the overall sample size. Mouse sizes (Table 2) and activity concentrations in the various organs/tissues were randomly chosen for each simulation with a normal probability based on typical biodistribution studies reported in the literature [5]. The sizes of simulated mice were read from the output log file of the Moby software, whereas the volumes were calculated as described earlier for the experimental studies.

**Table 2:** Physical characteristics of mice used in the simulated group

		Minimum	Maximum	Mean	SD
Body dimensions (mm)	Cephalad-caudad	87.270	91.340	88.980	1.050
	Ventral-dorsal	15.150	21.710	19.120	2.090
	Lateral	26.440	34.480	29.120	2.400
Volume (ml)	Brain	0.297	0.511	0.401	0.062
	Heart	0.154	0.277	0.209	0.033
	Lungs	0.412	0.724	0.548	0.082
	Kidneys	0.204	0.354	0.273	0.042
	Bladder	0.038	0.068	0.052	0.008
	Liver	1.111	1.930	1.492	0.230
	Pancreas	0.215	0.376	0.289	0.045
	Spleen	0.066	0.109	0.084	0.012
	Stomach	0.316	0.545	0.419	0.062
	Testis	0.196	0.337	0.259	0.038
	Skeleton	1.041	1.708	1.350	0.189
	Remaining organs & skin	14.203	24.250	18.726	2.818
	Total	18.253	31.187	24.101	3.618

**Fig. 3:**
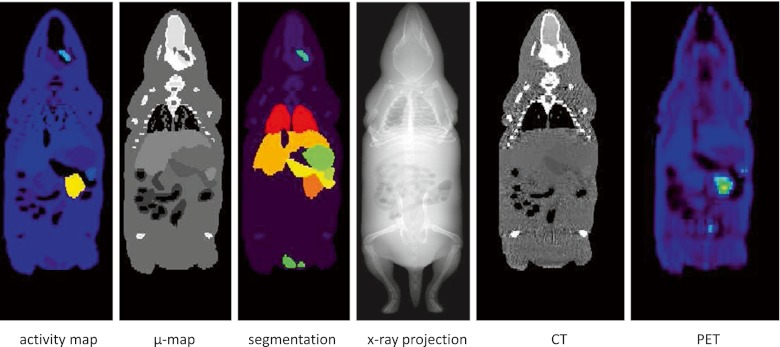
Coronal views of a voxel-based mouse model generated using the Moby software for simulation of an ^18^F-FDG study showing from *left to right*: activity map, attenuation map, segmented image, simulated X-ray projection image, simulated CT image and corresponding simulated PET image

The Moby software was also used to produce segmented images by modifying the input parameters to simulate tracer uptake in only one organ at a time, leading to an image for each segmented region/organ. All images were then combined using an exclusive operator to avoid having voxels labelled as two different organs, thus producing one unique image corresponding to the mouse segmentation serving as reference. This image is finally corrected by filling holes in the heart and lung regions using the ITK-SNAP software to simulate the Digimouse segmentation. An example of the three voxelized images is shown in Fig. 3.

We used the CT projector provided with the Moby mouse software to generate realistic mouse CT images corresponding to the simulated PET data [28]. The projector was used to obtain 360 projections, one per degree, with the characteristics of the cone-beam microCT device used in our laboratory [29]:

- Object to source distance = 22.2 cm
- Object to detector distance = 6.8 cm
- Half-fan angle = 19°
- Pixel size of 0.2 × 0.2 mm^2^

CT images were then reconstructed from the simulated projection data with a voxel size of 0.2 × 0.2 × 0.4 mm^3^ using Feldkamp’s algorithm [30] as implemented in the MATLAB image reconstruction toolbox developed by Dr. Fessler.^1^ PET images of the simulated Moby model were reconstructed with attenuation correction using the Software for Tomographic Image Reconstruction (STIR) package [31] by means of the ordered subsets version of Green’s maximum a posteriori (MAP) using the one-step late algorithm [32] with 24 subsets, 25 sub-iterations and inter-update Metz filtering [33]. Example images of X-ray projections and CT and PET reconstructed slices are shown in Fig. 3.

### Automated analysis and qualification

As explained earlier, the proposed approach is based on 3-D non-rigid image registration between actual mice (source or moving image) and the Digimouse atlas (target or fixed image). The main restriction of our method is that PET/CT images (as opposed to PET only images) of the moving mouse are required since the high-resolution anatomical CT image serves as the basis for actual mouse to atlas registration. The second constraint is that similar fields of view for source and target images have to be made available.

Following CT to CT image coregistration, the generated spatial transformation resulting from this procedure is used to transform the moving PET image. This will produce a new PET/CT data set of the actual mouse in the atlas space. Thereafter, atlas segmentation is used to automatically define VOI to measure the radiotracer’s activity concentration in various organs of the actual mouse. Therefore, the image registration process is the key point of this approach. For this reason, the methodology followed and the main parameters used as well as the algorithm’s performance assessment are described in detail below.

#### Image registration

Image registration was performed using the Elastix software suite [34]. The package consists of a collection of algorithms based on the Insight Toolkit (ITK) libraries that can be run from command line. One of the advantages of this software is that it enables saving the calculated transformation following the coregistration into an ASCII file that can be further used to transform other images using the same transformation parameters by means of the Transformix tool [34]. This software was conceived as a set of modules that can be parameterized individually depending on the needs of the user to handle particular situations. In our case, the following choices were made:

- All calculations were carried out with floating-point precision.
- Multi-resolution registration was performed at 5 levels with down-sampling factors of 32, 16, 8, 4, and 2 times, each with first-order B-spline image smoothing using 4,096, 4,096, 2,048, 2,048 and 1,024 iterations, respectively.
- Normalized correlation coefficient was used as the cost function metric since it was reported to perform slightly better (compared to mutual information) in intra-modality image coregistration [34].
- The image sampler used to compute the cost function is composed of 2,500 random points that were updated for each iteration.
- The adaptive stochastic gradient descent was used as the optimization method to adapt the step size sequence.
- The final interpolation is a third-order B-spline function.

The registration was performed in three steps:

1. *Affine registration:* mainly used to pre-align both CT images to facilitate the task of the non-rigid registration procedure. It is composed of 12 degrees of freedom, 3 for each of the possible transformations: translation, rotation, scaling and shearing.
2. *B-spline non-rigid registration:* applied to obtain a good alignment of CT images in size and shape. This step produces very good results for mouse shapes, but in most cases it does not suffice for internal organs. This transformation step allows an infinite number of degrees of freedom.
3. *Masked B-spline non-rigid registration:* applied to obtain a finer registration of internal organs. This step was added to improve the quality of image registration without spending considerable computational resources outside the mouse surface. We used one unique mask based on the Digimouse atlas shape since, based on previous registration steps, the actual mouse CT image now has the same shape.

Once the three steps are completed, we apply the same transformation parameters to PET images of the same mouse as well as its segmented image to obtain a PET/CT and segmented data set corresponding to the Digimouse space. The performance of the image registration algorithm was assessed using two well-established metrics [35]:

1. *Dice coefficient (D*_*coeff*_) *or mean overlap.* This is a volume overlap metric which quantifies the intersection between source and target regions divided by their mean volume:

1 $$ {D_{{coeff}}} = 2\frac{{\left| {S \cap T} \right|}}{{\left| S \right| + \left| T \right|}} $$

The Dice coefficient [36] is a special case of kappa statistical coefficient [37] which can be interpreted as follows:

- Less than 0.20 ➔ poor agreement
- 0.20 to 0.40 ➔ fair agreement
- 0.40 to 0.60 ➔ moderate agreement
- 0.60 to 0.80 ➔ good agreement
- 0.80 to 1.00 ➔ excellent agreement

2. *Hausdorff distance (HD*). This is the most frequently used discrepancy measure, which represents the maximum distance one would need to move the boundaries of the source region to completely cover the target region:

2 $$ H{D_{{S \to T}}} = \mathop{{\max }}\limits_{{s \in S}} \left\{ {\mathop{{\min \left\{ {d\left( {s,t} \right)} \right\}}}\limits_{{t \in T}} } \right\} $$

where *s* and *t* are points inside the source (*S*) and the target (*T*) regions, respectively, and *d(s,t)* is the distance between *s* and *t*. The measure of the directional Hausdorff distance *HD*_*S→T*_ is oriented and in most of the cases is not equal to *HD*_*T→S*_. For this reason, the generalized Hausdorff distance (*HD*) is defined as:

3 $$ \begin{gathered} HD = \max \left\{ {\mathop{{\max }}\limits_{{s \in S}} \left\{ {\mathop{{\min \left\{ {d\left( {s,t} \right)} \right\}}}\limits_{{t \in T}} } \right\},\mathop{{\max }}\limits_{{t \in T}} \left\{ {\mathop{{\min \left\{ {d\left( {t,s} \right)} \right\}}}\limits_{{s \in S}} } \right\}} \right\} \\ = \max \left\{ {H{D_{{S \to T}}},H{D_{{T \to S}}}} \right\} \\ \end{gathered} $$

#### PET normalized mean activity measurement

The mean activity concentrations in various regions of the PET images were calculated and normalized to the maximum activity concentration to obtain the normalized mean activities (NMAs). The NMAs from the pre-registered PET images prior to transformation for the seven segmented regions were used as reference. The automated PET analysis results are then compared to reference values by calculating the NMAs in the same seven regions based on the original Digimouse segmentation. The relative error between these two NMAs values is also calculated (in %).

## Results

The assessment of the accuracy of the image registration algorithm using well-established metrics is important because it will condition the automated activity quantification procedure. To avoid image segmentation bias, we have measured both metrics (*D*_*coeff*_ and *HD*) using the original Digimouse segmentation and our own segmentation of the Digimouse atlas, similar to the procedure followed for the segmentation of the experimental mouse sample. Statistical analysis performed to compare the means of all the regions using the paired *t* test for correlated samples is reported in terms of *p* values (0.05 was used as threshold for statistically significant differences). The analysis of the Dice coefficient revealed that the mean *D*_*coeff*_ was significantly improved (*p* < 0.0001) from 0.4557 to 0.5032 using our segmentation of the Digimouse CT image. However, the improvement of mean Hausdorff distance, from 6.33 to 6.16 mm, was not statistically significant (*p* = 0.143).

Similar to the procedure followed for assessment of image registration, we have tested if our segmentation of the Digimouse improved the results of the NMA analysis compared to the original Digimouse segmentation. This was performed by comparing the relative difference between quantitative estimates in the original and spatially transformed images using both segmentations of the Digimouse atlas, namely the original segmentation and our own segmentation. The *t* test for correlated samples revealed that the slight improvement of the mean relative difference (from 32.1 to 30.9 %) was not statistically significant (*p* = 0.214) when using our segmentation approach. Considering that only the Dice coefficient is influenced by the segmentation, we hypothesize that both Digimouse segmentations are equivalent and will not bias the results. For this reason, the results shown below are all calculated using the original Digimouse segmentation.

**Fig. 4:**
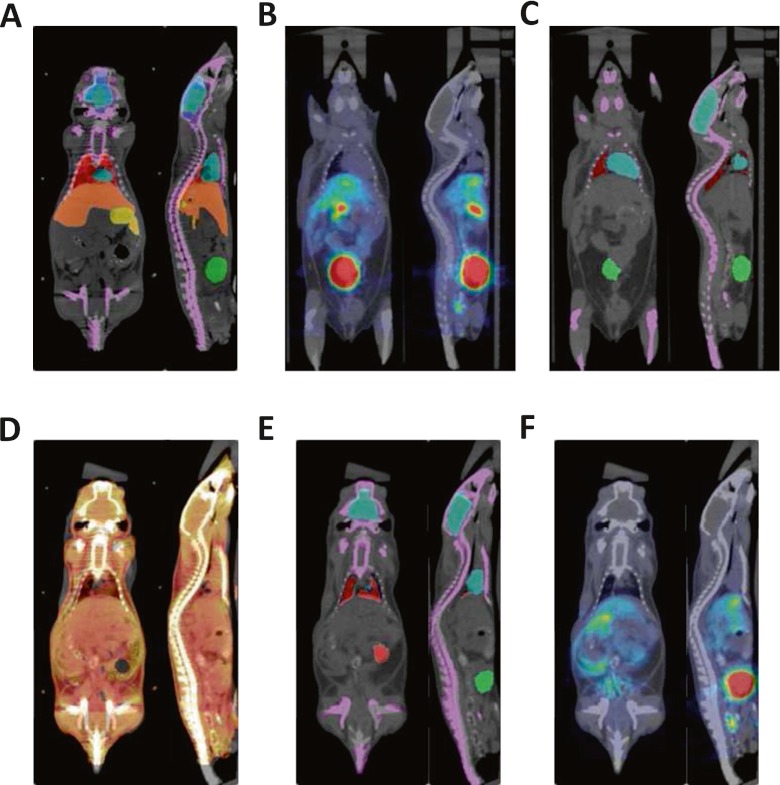
Illustration of the best deformable registration example between the Digimouse and one of the experimental mouse studies (mouse 4) showing: **a** overlay of the Digimouse atlas onto corresponding CT images, **b** actual ^18^F-FDG PET/CT mouse study, **c** mouse study shown in **b** with overlay of the segmentation onto CT image (seven organs), **d** CT to CT registration of the Digimouse and actual mouse study shown in **c**, **e** overlay of the transformed segmentation (seven organs) using registration parameters obtained in **d** onto CT image and **f** transformed PET/CT study using registration parameters obtained in **d**

**Fig. 5:**
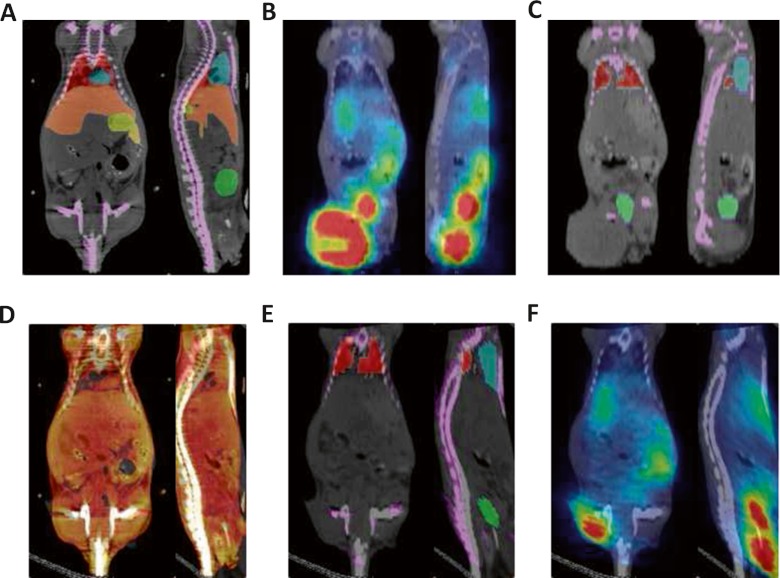
Same as Fig. 4 for the worst deformable registration example between the Digimouse and one of the experimental mouse PET/CT studies (mouse 6) acquired using ^68^Ga-labelled ethylenediaminetetraacetic acid (EDTA)

Typical image registration results between experimental mouse studies and the Digimouse atlas are shown in Figs. 4 and 5 as representative of the best (mouse 4) and worst (mouse 6) cases, respectively, considering the Dice coefficient as metric for assessment.

As discussed earlier, a large variability was expected for the experimental studies given the heterogeneity of the considered sample. To illustrate this variability, Table 3 summarizes the results for the Dice coefficient and the Hausdorff distance for all segmented regions of the experimental and simulated mice. One should note the large difference among Dice coefficients corresponding to best (0.594 ± 0.208) and worst (0.308 ± 0.276) registration results (Figs. 4 and 5).

**Table 3:** Summary of mouse registration results for the 8 experimental mice and 17 simulated mice studies when using the original Digimouse segmentation

Mouse		Dice coefficient		Hausdorff distance (mm)	
		Mean	SD	Mean	SD
Experimental	Mouse 1	.457	.284	6.41	3.46
	Mouse 2	.430	.275	7.55	5.13
	Mouse 3	.489	.271	7.02	5.15
	Mouse 4	.594	.208	5.24	3.83
	Mouse 5	.527	.247	5.10	2.92
	Mouse 6	.308	.276	6.83	2.17
	Mouse 7	.473	.251	5.62	2.88
	Mouse 8	.332	.306	6.88	1.74
Simulated	Moby 1	.445	.257	5.96	2.68
	Moby 2	.443	.261	6.04	2.70
	Moby 3	.445	.257	5.93	2.65
	Moby 4	.455	.258	5.97	2.68
	Moby 5	.444	.259	5.95	2.70
	Moby 6	.451	.263	5.93	2.75
	Moby 7	.453	.265	6.00	2.66
	Moby 8	.428	.268	6.25	2.77
	Moby 9	.455	.259	5.91	2.68
	Moby 10	.459	.263	5.93	2.82
	Moby 11	.449	.261	6.11	2.68
	Moby 12	.450	.260	5.98	2.76
	Moby 13	.451	.262	5.98	2.72
	Moby 14	.447	.262	5.95	2.66
	Moby 15	.461	.261	5.99	2.74
	Moby 16	.447	.263	6.11	2.71
	Moby 17	.408	.263	6.49	2.48

**Fig. 6:**
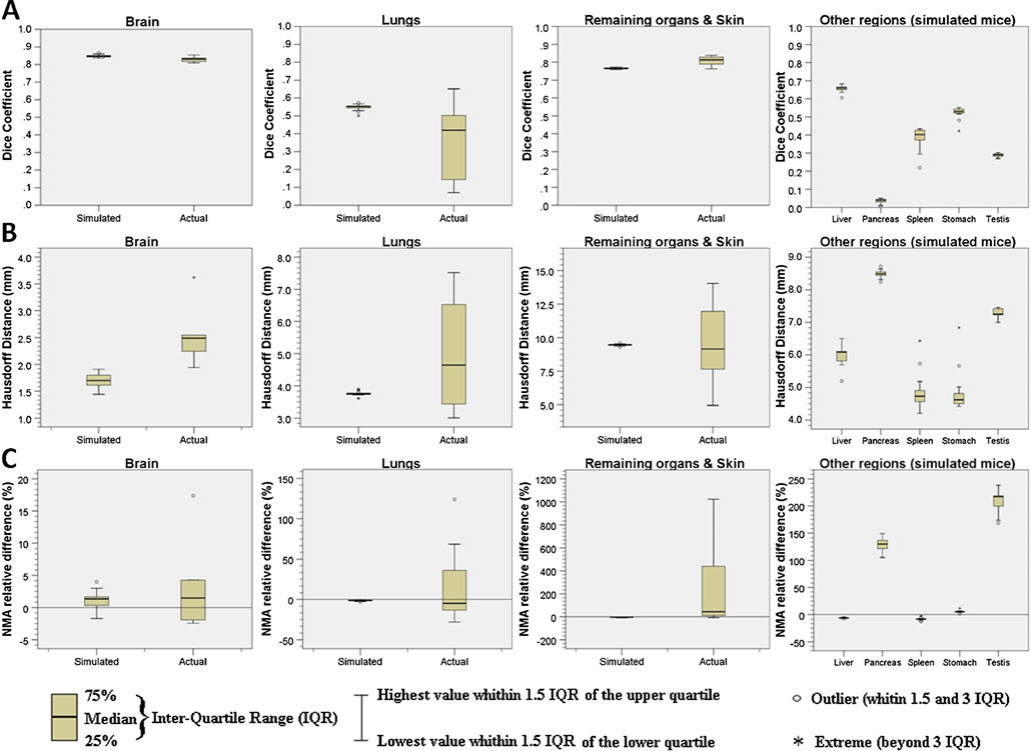
Box-and-whisker plots of image registration metrics (DC and HD) and automated quantitative analysis (NMA) results for the 8 experimental and 17 simulated mice studies for the 3 and 8 segmented regions, respectively. **a** Dice coefficients, **b** Hausdorff distance and **c** normalized mean activity (NMA) relative difference

Dice coefficient, Hausdorff distance and normalized mean activity estimates before deformation (prior to image registration) as well as the mean relative difference for the NMAs introduced by the automated quantitative analysis procedure for the seven segmented regions for both experimental and simulated mice studies are shown in Table 4. Some representative results of these metrics are presented in box-and-whisker plots for different regions corresponding to experimental and simulated mouse studies in Fig. 6.

**Table 4:** Summary of image registration performance metrics and normalized mean activity measurements of original mice (before transformation) and the relative difference between the original and the automated quantification procedure when using the Digimouse segmentation

Region	Type of study	Dice coefficient			Hausdorff distance (mm)			Normalized mean activity					
								Original (before transformation) quantification			Automated quantification relative difference (%)		
		Mean	SD	*p* value	Mean	SD	*p* value	Mean	SD	*p* value	Mean	SD	*p* value
Brain^a^	Simulated	.848	.008	0.081	1.70	0.13	0.029^c^	5.07E02	1.66E03	0.523	1.01	1.46	0.451
	Experimental	.827	.018		2.57	0.64		4.09E02	3.52E02		3.75	8.08	
Heart	Simulated	.536	.024	0.369	3.34	0.47	0.247	3.51E02	1.48E03	0.827	2.77	1.47	0.517
	Experimental	.485	.146		3.75	0.85		3.80E02	3.58E02		19.26	67.12	
Lungs	Simulated	.548	.018	0.046^c^	3.76	0.08	0.097	4.11E02	1.83E03	0.049^c^	-1.37	1.00	0.378
	Experimental	.357	.213		4.97	1.72		2.12E02	2.24E02		16.43	52.38	
Kidneys	Simulated	.455	.019	0.076	4.01	0.30	0.288	1.23E01	6.79E03	<0.0001^c^	-34.54	1.25	0.040^c^
	Experimental	.387	.086		4.48	1.05		2.44E02	1.42E02		-8.10	28.20	
Bladder	Simulated	.000	.000	0.045^c^	9.42	0.25	0.051	5.22E01	2.07E02	0.158	-94.35	0.22	0.064
	Experimental	.185	.230		6.80	2.99		3.98E01	2.13E01		-64.57	36.83	
Skeleton^a^	Simulated	.313	.008	0.202	9.23	0.13	0.231	3.23E02	1.23E03	0.492	3.70	1.37	0.179
	Experimental	.280	.064		10.76	3.20		5.25E02	7.75E02		-3.37	12.88	
Remaining organs & skin^b^	Simulated	.766	.004	0.005^c^	9.45	0.07	0.927	3.82E02	1.64E03	0.037^c^	-4.83	1.28	0.100
	Experimental	.808	.027		9.56	3.16		1.79E02	2.12E02		250.27	367.46	

^a^In three of the eight experimental mouse studies, the brain region is absent and the skeleton region is truncated

^b^The definition of the “remaining organs & skin” region is not the same between experimental and simulated mouse studies

^c^Regions where there are statistically significant differences (*p* < 0.05) between experimental and simulated sample means

Paired *t* test analysis for independent samples was performed to test the null hypothesis that mean values (*D*_*coeff*_, *HD*, *NMA* before transformation and automated *NMA* relative difference) obtained with the experimental mouse sample is different from the mean value obtained with simulated mice. One can see that statistically significant differences between experimental and simulated samples were found for lungs, bladder and remaining organs & skin regions for Dice coefficient, whereas this was only the case for the brain for Hausdorff distance. With regard to NMA before registration, one can observe that there is evidence of statistically significant differences between experimental and simulated mice activity concentration for kidneys, lungs and remaining organs & skin regions, probably because three different radiotracers were utilized for the experimental mice sample, whereas only FDG was used for simulated studies using the Moby model. Finally, a statistically significant difference is observed only for the kidneys between experimental and simulated mouse sample results.

Based on the foregoing statistical analysis, we can in some cases unify experimental and simulated studies into a unique sample which will be more representative of the performance of the developed automated quantification method. The results of this unified analysis are shown in Table 5, where two estimates are shown for regions where there is a statistically significant difference between experimental and simulated studies. The 95 % confidence interval was also calculated taking into account the sample size.

**Table 5:** Summary of the pooled performance evaluation of the automated coregistration procedure and quantitative analysis of PET data using the 95 % confidence interval

Region	Dice coefficient	Hausdorff distance (mm)	Normalized mean activity	
			Unmodified manual quantification	Automated quantification relative difference (%)
Brain^a^	.844 ± .006	1.70 ± 0.07^c^	4.85E02 ± 0.71E02	1.63 ± 1.74
		2.57 ± 0.79^d^		
Heart	.520 ± .035	3.47 ± 0.25	3.60E02 ± 0.80E02	8.04 ± 15.29
Lungs	.548 ± .009^c^	4.14 ± 0.45	4.11E02 ± 0.08E02^c^	4.33 ± 12.17
	.357 ± .178^d^		2.12E02 ± 1.86E02^d^	
Kidneys	.433 ± .024	4.16 ± 0.27	1.23E01 ± 0.34E02^c^	-34.52 ± 0.64^c^
			2.44E02 ± 1.18E02^d^	-8.10 ± 23.53^d^
Bladder	.000 ± .000^c^	8.58 ± 0.84	4.82E01 ± 0.54E01	-84.82 ± 10.06
	.185 ± .192^d^			
Skeleton^a^	.302 ± .016	9.72 ± 0.77	3.87E02 ± 1.77E02	1.44 ± 3.22
Remaining organs & skin^b^	.766 ± .002^c^	9.49 ± 0.70	3.87E02 ± 0.08E02^c^	76.80 ± 95.86
	.808 ± .022^d^		1.79E02 ± 1.77E02^d^	
Liver	.657 ± .009	5.97 ± 0.15	5.23E02 ± 0.11E02	6.12 ± 0.60
Pancreas	.036 ± .006	8.49 ± 0.06	1.89E02 ± 0.04E02	-129.80 ± 5.75
Spleen	.387 ± .029	4.84 ± 0.28	4.93E02 ± 0.13E02	8.17 ±1.23
Stomach	.524 ± .016	4.82 ± 0.31	3.65E02 ± 0.08E02	-5.37 ± 1.06
Testis	.289 ± .005	7.30 ± 0.06	3.60E02 ± 0.06E02	-208.50 ± 10.05

^a^In three of the eight experimental mouse studies, the brain region is absent and the skeleton region is truncated

^b^The definition of the “remaining organs & skin” region is not the same between actual and simulated mouse

^c^Simulated mice mean values with 95 % confidence interval

^d^Experimental mice mean values with 95 % confidence interval

A similar approach to the one adopted by Kesner et al. [5] was used to assess the accuracy of the automated analysis algorithm, which consists in measuring the so-called harvested standardized uptake value (hSUV) that reflects the actual measurement of the activity concentration in dissected organs of the mice. This metric is then correlated through linear regression to VOI-based analysis. The accuracy of the automated quantitative method is evaluated using the correlation coefficient (*R* ^2^) of this regression.

By analogy, we define the harvested NMA (hNMA) metric as the activity concentration used as input in the voxelized Moby phantom, which is correlated to the activity concentration estimated using the automated analysis technique through linear regression analysis. Table 6 shows the mean correlation coefficient calculated for all organs for each individual mouse, whereas Table 7 shows the mean correlation coefficient calculated with all mice for each individual organ. The results obtained by Kesner et al. [5] using their semi-automated algorithm are also shown in both tables. It can be seen that the accuracy of our fully automated approach is comparable to that of Kesner et al.’s semi-automated method.

**Table 6:** Correlation coefficients (*R* ^2^) resulting from linear regression analysis between actual (harvested) and PET-derived activity concentration for simulated studies. The results obtained by Kesner et al. [5] for experimental studies using manual and semi-automated software approaches are also shown

Automated (this work)		Semi-automated Kesner et al. [5]		
			Software	Manual
Moby 1	0.954	Mouse 1	0.943	0.083
Moby 2	0.961	Mouse 2	0.957	0.973
Moby 3	0.950	Mouse 3	0.947	0.956
Moby 4	0.952	Mouse 4	0.893	0.825
Moby 5	0.965	Mouse 5	0.908	0.560
Moby 6	0.800	Mouse 6	0.999	0.276
Moby 7	0.797	Mouse 7	0.934	0.915
Moby 8	0.879	Mouse 8	0.483	0.424
Moby 9	0.764	Mouse 9	0.996	0.992
Moby 10	0.961			
Moby 11	0.767			
Moby 12	0.832			
Moby 13	0.842			
Moby 14	0.749			
Moby 15	0.890			
Moby 16	0.796			
Moby 17	0.749			
Mean ± SD	0.859 ± 0.084		0.896 ± 0.159	0.667 ± 0.341

**Table 7:** Correlation coefficients (*R* ^2^) resulting from linear regression analysis between actual (harvested) and PET-derived activity concentration for different regions of simulated mouse studies. The results obtained by Kesner et al. [5] for experimental studies using manual and semi-automated software approaches are also shown

	Automated (this work)	Semi-automated Kesner et al. [5]	
		Software	Manual
Heart	0.526	0.579	0.358
Brain	0.309	0.331	0.237
Lung	0.677	0.799	0.303
Liver	0.489	0.589	0.192
Spleen	0.229	0.038	0.193
Kidneys	0.584	0.636	0.513
Stomach	0.432	NA	NA
Skin	0.490	NA	NA
Skeleton	0.299	NA	NA
Pancreas	0.441	NA	NA
Testis	0.033	NA	NA

## Discussion

Automated quantitative analysis of PET data potentially may play a pivotal role in large-scale clinical trials and longitudinal serial preclinical studies involving the acquisition of a large sample of small animal imaging data. This work proposes and evaluates the performance of an atlas-guided automated quantification algorithm using simulated and experimental studies. The performance of the image registration algorithm is the critical issue in our automated quantification approach. In the experimental sample, we noticed a large variability in the results reported for the various metrics evaluated. For example, Dice coefficient results vary between 0.308 and 0.594 (mean = 0.451) (Table 3). Similar variability was observed for the Hausdorff distance, with values varying from 5.10 to 7.55 mm (mean = 6.33 mm), with larger regions having larger Hausdorff distances (*HD*_*Heart*_ = 0.85 mm; *HD*_*Skeleton*_ = 10.76 mm) (Table 4). In contrast to the experimental sample, the performance of image registration was less widely dispersed for the simulated sample, with a dispersion of Dice coefficients between 0.408 and 0.461 but with a mean (0.447) close to the one achieved for the experimental sample. The same trend was observed for the Hausdorff distance metric (min. = 5.91 mm; max. = 6.49 mm; mean = 6.03 mm). The most probable reason for the difference in dispersion of results is the presence of very large morphological deformations on the experimental mice owing to the presence of tumour xenografts. Nevertheless, one can observe that, in the overall sample composed of simulated and experimental mice, a final moderate agreement was reached regarding the performance of the image registration procedure (*D*_*coeff*_ = 0.448), with a mean mismatch of approximately 6 mm.

The analysis of the image registration procedure on a region-by-region basis (Table 4) reveals again that the algorithm performs better when using simulated compared to experimental mice owing to the presence of tumour xenografts, except for the bladder and to a smaller extent remaining organs & skin, where Dice coefficients of experimental mice are higher than those of simulated mice. One can also see that the brain and remaining organs & skin regions registration show good to excellent agreement (*D*_*coeff*_ > 0.76) for both experimental and simulated samples. Poor agreement was obtained for time varying organs such as the bladder owing to periodic filling and emptying (for experimental and simulated mice) and small structures such as the pancreas (for the simulated sample) (Table 5). A fair to good agreement was found for the other regions (0.289 ≤ *D*_*coeff*_ ≤0.657).

The statistical analysis revealed that, in most of the cases, experimental and simulated samples can be combined to obtain a unified larger sample with a higher statistical power. Some exceptions with respect to Dice coefficient are the lungs, likely because of the presence of some poorly aligned cases, since the median shown in Fig. 6 (*D*_*coeff*_ = 0.420) is much closer to the simulated mice results. The second exception is the bladder region, though this was expected owing to the poor performance of the image registration procedure for this organ. The last exception applies to remaining organs & skin region, which presents a good to excellent agreement and where the difference in the image registration results is influenced by the way the regions of interest are defined. It should be noted that in the experimental mouse segmentation remaining organs & skin include liver, pancreas, spleen, stomach and testis regions, owing to the low soft tissue contrast, while in the simulated mouse these consist of independent regions. The only exception with respect to the Hausdorff distance is surprisingly the brain region.

Several studies have reported on various algorithms implementing deformable image registration of actual mice to an atlas. For instance, the method proposed by Baiker et al. [38] is based on manual extraction of the skeleton and lung from source and target mice to be registered with a hierarchical realistic model of the skeletal joint movement. This method achieved Dice coefficients comparable to those reported in this work for kidneys (0.48), liver (0.62) and heart (0.50) regions. Worse results were obtained for the brain (0.75), whereas better results were achieved for the lungs (0.65) and skeleton (0.5). The most remarkable result achieved is the skin correspondence (maximum mismatch below 3.5 mm) from mice that are in different positions. Wang et al. [39, 40] performed image registration of mouse CT images with the Moby phantom, Digimouse atlas and their own statistical atlas constructed using microCT images of 45 mice. The registration is performed only for the torso by manually segmenting high-contrast organs to be registered; low-contrast organ positions are estimated from this transformation. Dice coefficients corresponding to this method are particularly high following registration with the authors’ statistical atlas (*D*_*coeff*_ > 0.70 except for the spleen, *D*_*coeff*_ ≈ 0.45). However, when the registration is performed with the Digimouse atlas, the performance is similar to that achieved in this work for low-contrast organs such as the liver (0.68), spleen (0.38) and kidneys (left kidney ≈ 0.39, right kidney ≈ 0.61), while better results were achieved for high-contrast organs such as the lungs (0.75) and heart (0.68).

When analysing the performance of automated activity quantification, one can see that, similar to the image registration procedure, lower relative errors were obtained for the simulated sample except for the bladder, where the results reflect poor image registration. The same observations were made for the kidneys, probably because of the relatively high activity concentration for the simulated sample, where a small mismatch might result in a non-negligible reduction in the estimated activity concentration due to the small size of the region. Considering the statistical analysis results of the two samples, one can see that a statistically significant difference in the relative error was obtained only for the kidneys. Therefore, all remaining regions can be analysed as a pooled sample. Table 5 shows that in 7 of the 12 analysed regions the relative error of the automated quantification procedure is below 9 %. In particular, the relative error for the brain, lungs and skeleton is below 5 %. The kidneys’ activity concentration estimation error was estimated to be around 20 %, while larger errors were obtained for other regions owing to poor image registration, except remaining organs & skin” region, where an excellent image registration was achieved but with a correspondingly large relative error in terms of tracer uptake quantification (76.8 %). This result is highly influenced by large errors (∼250 %) associated with three experimental studies owing to the presence of tumour xenografts, where the tracer is taken up by one leg belonging to remaining organs & skin region. When deformable registration handles this large deformation, it significantly changes the shape and reduces the volume of the lesions, thus introducing a large difference in tracer uptake estimation. This also explains the high standard deviation in the NMA metric for this region (367 %), since it reflects the analysis of three mouse studies presenting with large distorting tumour xenografts while the remaining five mouse studies present a mean relative error of 20.1 % (SD of 25.7 %).

The comparison of the proposed automated quantification method results with those of Kesner et al. [5] shows that similar performance could be achieved using both methods (Tables 6 and 7). The main strength of our fully automated approach is that only minor preprocessing of the images is required to select the same field of view, while most other approaches rely on partially segmented images and/or the use of external fiducial markers.

### Conclusion

We propose a novel atlas-guided approach for automated quantification of small animal PET studies. To this end, preprocessing for adaptation of PET/CT images of the actual mouse study and atlas fields of view is required. Automated quantification is achieved through 3-D image registration between CT images of the actual mouse and the Digimouse atlas. The transformations achieving this are afterwards applied to corresponding PET images to put them in the Digimouse *image space*. The Digimouse segmentation is used to define the regions of interest for quantitative analysis.

The method proved to provide similar performance compared to other proposed techniques requiring user intervention [5, 39, 40]. Normalized mean activity estimation was preserved between reference and automated measures in most of the regions considered, with relative errors below 10 %. The only regions resulting in higher relative errors are those corresponding to small organs presenting a large variability among mice such as the testicles, bladder, kidneys and pancreas. This can also be explained by physiological respiratory motion, cardiac motion and periodic filling and emptying of the bladder. This was, however, expected from image registration assessment results. Further refinement of the latter procedure is underway.

## Footnotes

1
http://www.eecs.umich.edu/∼fessler
